# Thirty shades of truth: conspiracy theories as stories of individuation, not of pathological delusion

**DOI:** 10.3389/fpsyg.2013.00406

**Published:** 2013-07-09

**Authors:** Marius H. Raab, Stefan A. Ortlieb, Nikolas Auer, Klara Guthmann, Claus-Christian Carbon

**Affiliations:** Department of General Psychology and Methodology, University of BambergBamberg, Germany

**Keywords:** conspiracy theories, narrative construction, personality science, individual differences, external validity, regulation, psychological methods

## Abstract

Recent studies on conspiracy theories employ standardized questionnaires, thus neglecting their narrative qualities by reducing them to mere statements. Recipients are considered as consumers only. Two empirical studies—a conventional survey (*n* = 63) and a study using the method of *narrative construction* (*n* = 30)—which were recently conducted by the authors of this paper—suggest that the truth about conspiracy theories is more complex. Given a set of statements about a dramatic historic event (in our case 9/11) that includes official testimonies, allegations to a conspiracy and extremely conspiratorial statements, the majority of participants created a narrative of 9/11 they deemed plausible that might be considered a conspiracy theory. The resulting 30 idiosyncratic stories imply that no clear distinction between official story and conspiratorial narrative is possible any more when the common approach of questionnaires is abandoned. Based on these findings, we present a new theoretical and methodological approach which acknowledges conspiracy theories as a means of constructing and communicating a set of personal values. While broadening the view upon such theories, we stay compatible with other approaches that have focused on extreme theory types. In our view, accepting conspiracy theories as a common, regulative and possibly benign phenomenon, we will be better able to understand why some people cling to immunized, racist and off-wall stories—and others do not.

## Introduction

“Superstition is actually a symptom of enlightenment, whoever is superstitious is always, […] much more of a person; and a superstitious society is one in which there are many individuals and more delight in individuality”

(Nietzsche, 1882/1974, p. 96).

So far, many psychological studies on conspiracy theories have confined themselves to a simple—yet often misleading—paradigm: The assumption that a clear distinction between an official truth and delusive idiosyncratic explanations can be made, and that supporters of conspiracy theories must hence be considered as individuals who have lost touch with reality and are in need for clear-cut explanations. Unfortunately, this reproach of oversimplification also applies to the methods commonly used to investigate conspiracy theories: The frequent use of questionnaires implies that conspiracy theories can be reduced to simple statements and that recipients of conspiracy theories can be seen as passive consumers who can be “diagnosed” by specific items.

How do these basic assumptions account for the vast majority of conspiracy theories emerging from the highly interactive sphere of the new media? Why are conspiracy theories about 9/11 far more complex and disquieting than the official version if they are supposed to provide simple answers? Why are contradictory explanations for Princess Diana's “disappearance” deemed equally plausible (see Wood et al., 2012)? This contrasts Goertzel (1994) who noted that conspiracy thinkers “offer the same hackneyed explanation for every problem” (p. 741), and not several contradicting explanations for one discrete event. And finally: Why are conspiratorial plots nearly omnipresent in contemporary literature, in movies and on television? The entertainment value of conspiracies should also be taken into account when explaining the unsolicited, excursive, and mutating dissemination of such theories.

It seems that research on conspiracy theories has often emphasized cognitive peculiarities of people who adhere to conspiracy theories, suggesting that believers in conspiracy theories are specific cases who have not much in common with the majority of people. As such, the ordinary actor is often a blind spot of current research, as has recently been pointed out by Sapountzis and Condor (2013).

In sum, we feel that it is time to leave the beaten track and to acknowledge conspiracy theories as a vibrant phenomenon of popular culture which reflects far more than pathological delusions or xenophobic attitudes. Inspired by the ground-breaking work of Timothy Melley (2000) we interpret the increasing popularity of conspiracy theories as an attempt to emphasize a personal set of values and thus to organize and regulate one's life experience in a meaningful way. According to Melley (2000), the general motif behind conspiracy theories is to emphasize the values of autonomy and individuality by inducing an intensive fear of being controlled by concealed external forces. For this state of mind Melley has coined the term “agency panic.” By suggesting that our personal freedom is at stake, conspiracy theories create awareness for the (potential) threats to human autonomy and individuality. At this point we transcend Melley by stating that the self-affirmative mechanism behind conspiracy theories should work for any set of values a person wishes to emphasize (e.g., freedom of speech, integrity of the traditional family, mental and physical health, etc.). Based on this hypothesis, we interpret the widespread doubt in an official truth and the great popularity of conspiracy theories as a crisis of ideologies that goes hand in hand with a crisis of individuality. Especially in pluralistic Western societies where the “grands récits” (Lyotard, 2005) of the past have lost their credibility, conspiracy theories can help to express and to share an individual system of values. When there is no generally accepted frame of reference any more, individuation is in need for alternative explanations.

From our point of view empirical studies on conspiracy theories have so far neglected the creative potential, the dynamic, the interactive, and the narrative qualities of conspiracy theories. The predominant paradigm of psychological research in the field of conspiracy theories assumes that a clear distinction between an official truth and delusive idiosyncratic explanations can be made. For instance, Lewandowsky et al. (2013) showed that taking the moon landing for a hoax is correlated with a disbelief in climate change and a rejection of the fact that smoking causes lung cancer. One can either believe that smoking causes lung cancer, or one might not. Furthermore, as the authors point out, this is not a question of belief in the first place; there is overwhelming scientific evidence for negative side effects of smoking. To deny them means to negate the validity of scientific knowledge in general. We deem it questionable that doubts about the reasons for the invasion of Iraq should generally be explained by the same cognitive mechanisms.

Belief or disbelief in theories of conspiracy has been examined by reducing stories to simple statements (e.g., “9/11 was an inside job”) that may serve as questionnaire items. First and foremost, these items are designed to meet the psychometrical requirements of questionnaire construction. Naturally, such questionnaire items cannot reflect the complex and diverse narrations entwined around ideas of conspiracy. We see dangers in applying this approach to investigating conspiracy theories: Without a psychological model, one can merely speculate which latent variable or construct was measured after all. It gets hard to distinguish possible facets of a trait—a supposed predisposition to accept conspiracy theories—*ex post* without such a model. Goertzel (1994) has already pointed to the weakness of questionnaire data when it comes to people's belief systems.

For instance, Swami et al. (2010) were able to explain 53.1% of variance in “9/11 Conspiracist Beliefs” with a structural equation model including personality variables. Importantly, “General Conspiracy Beliefs” accounted for only 14.4% of variance. Wagner-Egger and Bangerter (2007) tried to identify predictors for belief in two types of conspiracy theories: Conspiracy theories that accuse minorities (Type A) and conspiracy theories blaming authorities (Type B). No less than 18 personality constructs were included. Regression analysis showed that these constructs only accounted for less than 10% of variance in terms of Type A theories (*R*^2^ = 0.09), respectively, 16% of variance in terms of Type B theories (*R*^2^ = 0.16). Although these studies have clearly delivered important insights, up to 90% of variance is left unexplained.

Based on these findings and on our own questionnaire studies, we doubt whether these procedures are able to grasp the appreciation and fascination of such theories in full. The low to intermediary values of explained variance not only indicate that the approaches did not cover some important factors. We also do not know if a participant has merely adopted some overheard notions; or if he or she has arrived at a conspiratorial belief after time-consuming, extensive research. We also do not know if the conspiratorial belief is stable, or if new information would be regarded and integrated; if it is a merely personal opinion or if the believer is eager to share his or her view with others; and finally, if the belief would be guiding the person's actions, e.g., if he or she would engage in political activities.

Apart from some recent studies—e.g., Sapountzis and Condor (2013) have evaluated the spontaneous use of conspiracy narratives in interviews of Greek citizens and Lewandowsky et al. (2013) investigated conspiracist ideation in the blogosphere—most studies have focused on the recipients of conspiracy theories using questionnaires and drawing an artificial red line between believers and disbelievers. To our understanding, this approach reveals some misleading basic assumptions about conspiracy theories: (a) Conspiracy theories are treated as invariant entities (b) that can be reduced to single statements and (c) that recipients of conspiracy theories can be regarded as passive consumers and (d) that believers are always believers independently from the “quality” of the regarding storyline. By contrast, we argue that a majority of conspiracy theories emerge from the highly interactive sphere of the new media. Today, millions of people around the world create, compile, discuss, and reproduce conspiracy theories on internet platforms, private websites, or blogs. This relentless process of creation, modification, and serial reproduction blurs the classic difference of a distinction of production (*sender*) and recipient. If our assumptions hold, people should—when given the chance—construct a wide variety of stories, differing greatly in conspiratorial characteristics. Questionnaires are hardly able to capture the narrative process of acquisition, compilation, and reproduction in an ecologically valid way. Consequently, we suggest the method of *narrative construction* as a new means to explore the multi-facet phenomenon of conspiracy theories. This method allows an individual to construct their own *story* for a given event like 9/11 from a set of pre-defined pieces of information.

If a conspiracy theory is a dynamic narration reflecting an individual's values—built around a dramatic historic event—there should be a plethora of different theories, not only concerning the story's nucleus, i.e., the historic event. The variety of personality should, according to this assumption, lead to an evenly manifold variety of conspiracy theories. Additionally, if it was a prevalent method of identity shaping, almost everyone should be prone to construct a conspiracy theory. We tested these assumptions empirically.

### The present paper

In the first section, we shortly describe a study that sought for a link between cognitive self-efficacy and the belief in common conspiracy theories—yet yielded no results. Subsequently, the method of *narrative construction*^1^ is described. It was applied in a study with 30 participants. In the following section, we present the results of this study. Finally, we outline a theoretically framework which accounts for our findings and allows for an integration of other explanatory approaches. We then outline the common ground of our and other models and close with a short consideration of the dangers of conspiracy theories.

## Methods

In our first study on conspiracy theories, we followed the established research paradigm: A standardized questionnaire was applied to investigate the relationship between self-efficacy and belief in conspiracy theories. We shortly describe this study—that yielded no positive results—before we illustrate the method of narrative construction in detail.

In accordance with the premise that supporters of conspiracy theories share some kind of cognitive or emotional disposition, we expected people with a low level of self-efficacy to be more susceptible for any kind of conspiracy theory than people who reported a high level of self-efficacy.

### Method

Our standardized questionnaire comprised the German version of the *General Self-Efficacy Scale* by Schwarzer and Jerusalem (1995). We modified some items to emphasize the cognitive component of self-efficacy. For example, the item “Thanks to my resourcefulness, I know how to handle unforeseen situations” was changed to “Thanks to my resourcefulness, I know how to interpret unforeseen situations.” In addition, a scale was designed for the assessment of endorsement in conspiracy theories. The scale consisted of 10 items. For each item, the gist of a popular conspiracy theory was condensed into a statement (e.g., “The terrorist attacks of 9/11 were planned and executed by the American government.”). The participants were asked to rate the plausibility of each statement on a five-point Likert-scale ranging from 1 (“very implausible”) to 5 (“very plausible”).

### Sample

Twenty-two males and 41 females participated in this study. The sample included students, workers and senior citizens. The age of the participants ranged from 18–76 years and the average age was 29.6 years (*SD* = 13.3).

### Results

The relation between self-efficacy and belief in conspiracy theories turned out to be non-significant, Pearson's *r* = −0.04, *p* = 0.73, *n.s*. There was no pattern to be found, neither linear trends between variables nor higher-order relations by mere inspection of plotted data. The analysis of particular items and *ex-post-facto* attempts (splitting the sample by gender, by age, etc.) yielded no results.

### Discussion

The data did not justify—or even suggest—the assumption that self-efficacy is related to endorsement in common conspiracy theories. Nevertheless, this finding is relevant. These results are well in line with the results of a study by Wagner-Egger and Bangerter (2007) which examined the link between locus of control and belief in conspiracy theories. The authors reported a low inter-correlation between externality ratings and belief in a particular type of conspiracy theories which accuses minorities (*r* = 0.15; *p* < 0.05). These findings clearly challenge the basic assumption that supporters of conspiracy theories must be considered as helpless individuals in need for clear-cut explanations. Left with no direction how to refine the hypothesis or the questionnaires, we decided to develop a new approach for exploring conspiracy theories

#### Thirty shades of truth: the method of narrative construction

To analyze the phenomenon of conspiracy theories in an ecologically more valid way, we developed the method of *narrative construction*. Given a set of statements about an important event of contemporary history, people begin to build a narrative that is, for the most part, neither a pure official nor a clear conspiracy theory. Instead, people construct their idiosyncratic “shade of truth.” A more detailed description of this method can be found elsewhere^1^. In the present paper, we focus on a different aspect, but give a short account of material and procedure so the present paper is coherent and understandable.

To test our hypotheses that conspiracy theories are frequently occurring and that they are diverse and idiosyncratic stories built around an important event, we developed the method of “narrative construction.” Participants are provided with a deck of cards, each card bearing a statement related to a specified event (in our case 9/11). The deck was built to represent conspiracy-specific categories we had generated before with an inductive procedure. For each “fact,” there was one version (card) holding an official/canonical claim, one version bearing a mildly conspiratorial allusion, and one version holding a claim only compatible with an extreme conspiracy.

### Material for a narrative construction

To identify the typical constituents of conspiracy theories, we questioned 38 people to tell us which conspiracy theories they know of; and afterwards asked them to describe their favorite theory in detail. Subsequently, we asked “which elements are part of most conspiracy theories” as an open question. The answers were categorized by other interviewers; the resulting categories had to be defended in a discussion, as described by Mayring (2005), until all interviewers had agreed on a set of six categories for “elements of conspiracy theories,” including category definitions. The bottom-up generated items are *odd event, evidence, non-transparency, publicity, group of conspirers*, and *myth*. A more detailed description of this study and its results can be found elsewhere^1^.

We compiled 14 subsets for the deck of cards. With respect to the bottom-up derived elements of conspiracy theories: two for *group of conspirers*, one for *non-transparency*, one for *publicity*, three for *odd event*, three for *evidence*, and one for *myth*. Subset group consisted of three items (i.e., 3 cards) fueled with contents from typical (1) *official*, (2) *limited conspiratorial*, and (3) *unlimited conspiratorial* viewpoints. The *official* card always bore a category-related statement that was in accordance with official 9/11 reports and documents (drawing on respectable sources, e.g., governmental reports made public on the internet). For example, an official *group of conspirers*-item was: “9/11 masterminds were Islamist terrorists, led by Osama bin Laden, to attack the detested Western culture.”

The *limited conspiratorial* card was prepared with an item that contained an explanation describing a conspiracy of moderate strength. Specifically, this level was formed in accordance with Lutter's (2001) categorization of conspiracies, corresponding to a conspiracy limited in time and space. This can also be thought as matching 9/11-view “let it happen on purpose” (“LIHOP” in the terminology of Ganser, n.d.). In this view, the Bush administration did not initiate the attacks but knew beforehand and did not take countermeasures. We compiled information from web resources like Wikipedia that matched this level. The “group of conspirers”-item here read: “The US administration had let happen the 9/11 attack to justify the wars in Afghanistan and Iraq.”

The *unlimited conspiratorial* card assumed a conspiracy with no clear bounds within space and time, or a “make it happen on purpose” (MIHOP) viewpoint in the sense of Ganser (n.d.) For example, it read: “The US administration had planned and conducted the 9/11 attack to justify the wars in Afghanistan and Iraq.”

This three-level graduation was realized for each subset of cards. Finally, for each of the six categories (odd event, evidence, non-transparency, publicity, group of conspirers, and myth), there was at least one three-part subset of cards (one card with an official statement, one limited conspiratorial, and one unlimited conspiratorial).

Additionally, we compiled a triplet of cards where all statements were completely off-wall:

– The group “Scholars for 9/11 truth” assumes that energy weapon fire, by killer satellites from outer space, led to the World Trade Center collapse.
– The former officer of nuclear intelligence and author Dimitri Khalezov postulates that the Twin Towers as well as building No. 7 were brought down by underground thermo-nuclear devices.
– The Syrian newspaper Al-Thawra has reported that 4000 Jewish WTC employees were warned beforehand and did not show up on work on 9/11.

The resulting 42 cards—13 canonical statements, 13 statements alluring to a limited conspiracy, 13 extremely/unlimited conspiratorial statements, and 3 off-wall assumptions—were printed on cards (each around 10 × 6 cm; serif typeface, 12 pt. size, black letters on white ground) and laminated.

### Participants

Thirty persons (26 female, *M*_age_ = 22.4 years, range: 19–55 years) took part in the study. Some were students at the University of Bamberg and received course credit for participation; they were naïve to the aim of the study and had not been involved in any other study described in this paper. The participants were randomly assigned to two groups: (1) *modest contents group* and (2) *extreme contents group*. The first group received the off-wall, the canonical, and the limited conspiratorial items only. The extreme contents group was handed out the full set including the 13 unlimited conspiratorial items. The split-up was done to test a hypothesis not discussed in this paper.

### Procedure

The *modest contents group* was handed out a card deck with 29 items, containing all *official* and *limited conspiratorial* items (plus the three-card subset absurd). The *extreme contents group* received the same deck and additionally 13 *unlimited conspiratorial* items. All were asked to “construct a plausible story of the events of September 11th 2001, as a single coherent story or consisting of coherent or controversial fragments,” without time restrictions. When the participant had considered the story finished, the chosen items and their layout were written down. The participant was then asked to rate “how plausible the 9/11 story version just laid out is” on a five-point Likert-scale (among other questions related to other hypotheses). Overall, the participants spent 21 min on average to construct their story, with a range from 8 min to well over 30 min.

## Results

We will present quantitative data analysis first. In order to acknowledge the diversity in story content, we will then present three single cases, i.e., three individual theories about 9/11.

### Statistical analysis

We found no significant differences between groups with regard to the number of items taken from the off-wall set, tested by separate One-Way Analyses of Variance (ANOVA), *F*_(1, 28)_ < 1, *p* = 0.836, *n.s*.; with regard to self-rated plausibility of the story, *F*_(1, 28)_ < 1, *p* = 0. 451, *n.s*.; and with regard to the number of cards selected in total, *F*_(1, 28)_ < 1, *p* = 0.80, *n.s*. In the aspects relevant for the argumentation and discussion here the groups do not differ, so we will aggregate both samples to a single one. Detailed further analyses on the given data set can be found elsewhere^1^.

On average, participants used 14.80 statements/cards (*SD* = 5.47; range: 5–28 cards) to construct a story, 0.50 cards (*SD* = 0.86, range: 0–3) of them were from the off-wall set. The average self-rated plausibility was 3.90 (*SD* = 0.71, range: 2–5).

**Figure 1:**
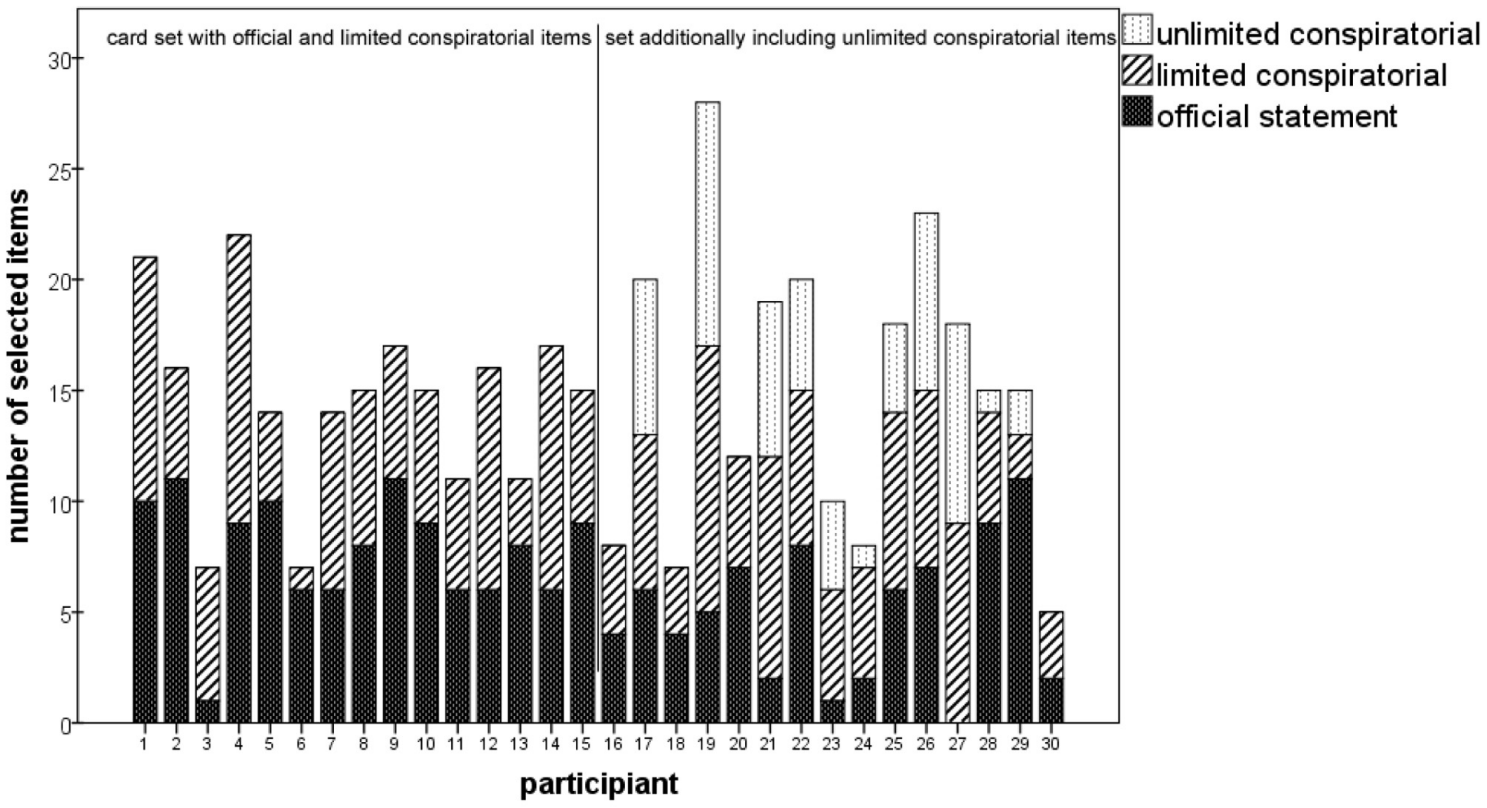
**Each participant created a unique story, blending official, limited, and unlimited conspiratorial items to build a plausible 9/11 narrative**.

There was a wide variety of length and content with regard to the theories produced. No two stories were alike; instead, highly idiosyncratic mixtures of statements were created. Figure 1 gives an impression of the diversity of compositions.

To reduce complexity and to test our hypotheses, we segregated the stories according to the share of official vs. limited and unlimited conspiratorial items. Here, we regard stories containing less than 33% conspiratorial items to be an “official version” of 9/11. Stories containing between 33 and 66% conspiratorial content were classified as a “hybrid version.” When more than 66% conspiracy-items were present, we considered the narrative as a “conspiracy version.”

With this deliberate categorization, 5 out of 30 stories (16.7%) qualified as official, 16 out of 30 stories as hybrid (53.3%), and the 9 remaining stories (30.0%) as conspiracy versions. We could neither detect a significant correlation between the self-rated plausibility and the number of off-wall items selected (*r* = 0.28, *p* = 0.88, *n.s*.) nor between plausibility and the number of items selected in total (*r* = −0.09, *p* = 0.62, *n.s*.). Regarding the content of the off-wall items, the killer satellites from outer space were present in three stories. Nine times, the thermo-nuclear devices were part of a story. The allegation of Jews knowing about the attacks beforehand was selected four times.

### A game of conspiracies: examples for 9/11 narratives

So far, we have analyzed only superficial information (e.g., number of items; composition of different item categories) of the generated versions. To understand the stories behind these numbers, we present three examples in detail (yet, each example is an abridged version; the full narratives were at least twice the length). We begin with a corner-case, the most canonical version that was produced. We proceed with a typical hybrid version that integrates many official statements as well as some propositions indicating a possible cover-up. Finally, we give an account of the most extreme conspiracy version that was built.

**A canonical story:** *On 9/11, four passenger planes got hijacked by Islamist suicide attackers; two of these planes were directed into the WTC twin towers. The resulting structural damage to the buildings led to their collapse. When President Bush was told about the second plane crashing into the towers, he kept sitting for five minutes—with countenance unaffected and seemingly not surprised—in front of the class at school, without interrupting the visit. On the day of the attacks, there was great confusion among the leading action forces. The chain of command expended too much time, as the US administration was not prepared for this kind of attack. Thus, the plane heading for the Pentagon could not be brought down in time. The 9/11 course of events was examined by several US agencies, supporting the official view. This was written down, for example, in the ‘9/11 Commission Report’.*

This is an abridged version of the only story (out of the sample of 30) that contains virtually no allegation to any conspiracy or cover-up. The originator, a 20-year-old woman, used seven items in total. Subsequently, she rated her story as most plausible (5 out of 5 points). The participant stated that she had “little interest on the issue of 9/11,” and that she had “recognized conspiratorial items,” but had discarded them as being “too speculative”; furthermore, she stated to have heard “about the unreliability of eye-witness in a lecture” some days before, and stated this might have made her “more cautious.”

**A hybrid theory:** *The 9/11 perpetrators had been Islamist terrorists under guidance by Osama Bin Laden to attack the hated “West”. Islamist terrorists had hijacked four passenger planes, two of which were directed into the twin towers. Standard operating procedures for hijackings were bureaucratic and chain of command operated slowly. There were lapses, failures, and precious time was lost. There should be further inquiries to clear up the countless open questions. The attack on Pearl Harbor was let happen by the US administration to get the own, war-weary people into WW II. Similar could have happened on 9/11.*

This is the abridged version of a typical hybrid story, constructed by a 25-year-old woman. The narrative combines official as well as mildly/limited conspiratorial items. While Islamist terrorists are identified as perpetrators, the possibility of a government letting happen the attack is included in the narrative. The creator of the story rated her story with 4 on the 5-point plausibility scale afterwards.

**A conspiracy theory:** *The US administration has initiated 9/11 itself, to justify the wars in Afghanistan and Iraq. There should be further inquiries to clear up the countless open questions. The US administration is lying. It was lying about the supposed nuclear weapons in Iraq, about Vietnam, Watergate, about many things. Why should one believe the ‘official’ 9/11 story? The WTC towers had been built with fire-resistant steel. The question remains: How could the towers had collapsed? The magazine Newsweek was reporting that high rank Pentagon officials had canceled flights scheduled for 9/11. According to the Syrian newspaper Al-Thawra, 4,000 Jewish employees did not show up at work on 9/11; they had been warned. There was and is one constant in the USA's policy: lie, deceit, and deception of her enemies and the public. This can be seen with Pearl Harbor, Watergate, the landing on the moon, and in recent times the 9/11 attacks*.

This is the abridged version of the story constructed by a 26-year-old woman. She was the only participant to include not a single item from the pool of official statements, using nine mildly/limited and nine extremely/unlimited conspiratorial items. Concerning plausibility, she rated her story with 4 afterwards.

## Discussion

The multiplicity in the content of conspiracy theories that was predicted by our assumption is clearly reflected by the obtained data. There was no dichotomy between official and conspiratorial; instead, we found “thirty shades of truth.” There was no restriction regarding the combination of items, and the statements stemmed from real-world sources and had not been fitted for representativeness (regarding the levels official, limited, and unlimited conspiratorial). We therefore cannot infer a strict rank ordering of the stories. Our deliberate trifold categorization must hence be considered a rough measure. Yet, regarding the shares of official and conspiratorial items, our data shows the tendency to construct conspiracy theories, although in most cases, moderate ones. Interestingly, the only strictly canonical story was deliberately constructed by a person that reported to have no great interest in the matters of 9/11.

Furthermore, in the short survey after the experiment, many participants stated that it was *fun* to compile an explanation for the events on 9/11, while the plausibility of the stories was high, assessed by ratings afterwards. In our view, this is a strong argument for ecological validity; it implies that people were engaged in a cognitive as well as an emotional way.

A possible limitation can be seen in the fact that we asked German people to construct a 9/11 narrative; for sure, a sample from the USA would yield other results. Yet, our goal was to induce active story construction, so we deliberately chose this topic: We could be sure every participant knew of this event; and at the same time we could be fairly sure there was no personal involvement—in a sense that a participant might have known one of the 9/11 victims personally.

The hypothesis that people with a low feeling of security are particularly prone to conspiracy theories—a hypothesis derived from the literature (e.g., Goertzel, 1994)—was not confirmed by the data of our first study. Statistical power was not sufficient to refute this hypothesis in general, but low self-efficacy at least does not seem to be a major factor. General racist beliefs as common drivers for conspiracies (e.g., Grüter, 2010) did not appear to be a relevant factor of influence with our sample, either. Only a minority (4 out of 30) chose to integrate the card claiming that Jews knew of the attacks beforehand into their storyline. The item explicitly ascribed the statement to a Syrian newspaper, so choosing it would have left a cognitive back door—the anti-Semitic allegation could be attributed to the source; hence, picking this card could be justified in the sense of “I do not believe it, but I believe that a Syrian newspaper wrote it.” The claim that thermo-nuclear devices had been mounted in the Twin Towers seemed to be more plausible, as it was chosen by nine participants.

In sum, two of the most common explanations for inter-individual differences in terms of conspiracy endorsement—need for security and racist attitudes toward minorities—could not be confirmed as driving factors. What, then, could be motifs to construct conspiracy theories? In the following sections we present a theoretical framework which accounts for the subtle “shades of truth” revealed by *narrative construction*, as well as the common deficit-oriented approaches focusing on extreme tendencies of conspiracy beliefs. This theoretical framework is based on the assumption that conspiracy theories are means to express personal beliefs and values, to relate these values to contemporary history, and to engage in discussions about values and agency.

Our method of narrative construction does not aim at measuring a latent variable as, by contrast, an intelligence test does. Instead, its purpose is to initiate a *process* in an ecologically valid way. Thus, we cannot determine reliability in the sense of classical test theory. Stability, as a special case of reliability, will have to be determined in a further study. If our hypothesis holds and conspiracy theories are a means of expressing one's personal values, this does not imply that a participant chooses exactly the same items on the following day. Nevertheless, it implies that the set of values reflected by this individual's stories should remain stable even over the course of months or years.

Our next step will be to address reliability in terms of stability, as it is crucial for our claims. A data-driven system of analysis will be designed to categorize the beliefs and values implied by a story. Moreover, participants will be asked to state their most important values explicitly. By employing a longitudinal design changes and invariants will be examined.

### The twilight of myth

The universality of certain narrative patterns and symbols has already been pointed out by Sigmund Freud and Carl Gustav Jung. They hypothesized that the exploration of myths—individual stories like dreams as well as universal ones—is a *via regia* for understanding the human psyche. Different academic disciplines try to fathom out the universality of myth and religion, emphasizing an interplay of nature and culture (Burkert, 2009) or cognitive operators that were shaped by evolution (Newberg et al., 2003). According to Bischof (1998), the structural universality of myths about world creation can be explained without assuming a collective unconscious. He argues that myths about world creation reflect the development of consciousness every individual experiences during early ontogenesis.

The existence of myths was a cultural constant and served to exemplify and consolidate group norms. The advent of the “self-expressive individual” (Campbell, 2008), however, rendered these myths meaningless and left the individual in the dark about desirable goals in life. With the beginning of enlightenment, assigning an individual his or her place in society by a story has begun to lose its importance. In return, the individual has to bear the burden of shaping society: “It is not society that is to guide and save the creative hero, but precisely the reverse. And so every one of us shares the supreme ordeal—carries the cross of the redeemer—not in the bright moments of his tribe's great victories, but in the silence of his personal despair” (Campbell, 2008, p. 337).

When stories, oral and written, have been the primordial and most important means to negotiate the relation between individual and society, we might assume that the means might prevail, even when the focus changes. It is desirable to know one's own motifs. At least, we deem it worthwhile to take this stance—and to see if it will be generative. Consequently, we will consider conspiracy theories as narrations that help people to recognize themselves, to define and express their system of values, and also to help them to articulate their demands on society. This does not necessarily imply that conspiracy theories are modern myths. In the first place, they are stories intertwined with defining society and ourselves; and successors of stories called myths, which had a distinctly different function, and distinctly different structural features.

A similar viewpoint was taken by Kelley-Romano (2008). She examined the television series *The X-Files* and concluded that the function of ubiquitous conspiracy in the series “defines what it means to be good or evil and simultaneously questions the process of identity formation itself” (Kelley-Romano, 2008, p. 106). Although the author recognized the psychological functions of the series' conspiratorial motives as crucial for its success, she did not describe the psychological parts of her theory in more detail.

Today, identity formation (at least in Western cultures) might be considered as the challenge to *shape* society *and* oneself. This does not necessarily have to be a painful process. For some, anxiety and a loss of control might be predominant, probably those of them who show a low degree of ambiguity tolerance in the sense of an “emotional and perceptual personality variable” (Frenkel-Brunswik, 1949). Some will meet this challenge with indifference. For others, it might appear as a playful and exciting endeavor to shape one's identity, probably on basis of the mere attempt to bring order into the story (see the “Aesthetic Aha” effect in Muth and Carbon, 2013), although a final solution might not be the ultimate source of reward (Muth and Carbon, 2013; Muth et al., in press). Embracing this full range might help to understand why conspiracy theories are not a well-separated niche of psychology and society—but, according to our data, pervasive.

### Shaping the pillars of the self

Dan McAdams assumed that “we are all tellers of tales” (McAdams, 1993) by the mere fact. Tales appear to him as a means to achieve self-insight, as a very basic way of organizing information—and a way to share this information, also about the coordinates of oneself within the society, in the world. McAdams integrates biological, developmental and cognitive aspects to explain why certain characters (like the Teacher, the Warrior, the Maker, the Friend, and the Survivor) frequently appear in such stories. During adolescence certain questions arise. For instance: “What is good? What is true? What is beautiful? How does the world work? How should the world work?” (McAdams, 1993, p. 82) The benefit of stories for self-awareness, their potential to render non-conscious ideas and values explicit, is also emphasized by Wilson (2002).

Right after puberty, stories like legends and myths are replaced by “theories and creeds and other systematic explications” (McAdams, 1993, p. 85). Such theories offer the opportunity to define the goodness (and badness) of very specific actions, and to evaluate them. The acquired belief and value system is likely to stay—with changes in detail—for the rest of one's lifetime forming the basis for the story that reflects and forges our self in adulthood.

It is noteworthy that a theory “impressively differentiated and integrated” (McAdams, 1993, p. 90) might be considered “particularly mature, advanced and enlightened” (p. 90). McAdams did not have conspiracy theories in mind; from a formal point of view, however, conspiracy theories also match his criteria. Particularly, the high degree of differentiation is one of the most striking features of conspiracy theories. We also observe that several story parts of a conspiracy theory are imperatively held together, at least by *ad-hoc* explanations or flexible interpretations of several parts toward a coherent Gestalt. This is also an important difference between a conspiracy “theory” and a truly scientific theory—the first one might be driven and put together by scientifically invaluable arguments but will yield a story which attracts people and which invites to fill the logical gaps by own considerations. This will raise the mere consumer to the position of the narrator and the creator her/himself.

The shift from society to the individual when it comes to defining values, however, should not be seen as a burden alone. Gergen (2006) sums up a debate about the consequences of emphasizing the distinction between oneself and the others: Becoming an individual implies the danger of isolation and alienation. Melley (2000) makes a similar point by hypothesizing that a certain amount of paranoia is not only a defense, but even a part of liberal individualism.

In *La condition postmoderne*, Jean-Francois Lyotard claims that the collapse of the grand narratives does not necessarily imply an atomization of society. Being part of a fabric of relations, even “the most underprivileged self” (Lyotard, 2005, p. 55) is not powerless in the language games of global communication. The self can treat messages as if it were the sender, the receiver, or the relator (Lyotard, 2005). Likewise, a conspiracy theory is an invitation to receive information, to share information, and also to add new information, in the end: to be a part of the generation and evolution process of a story.

Thus, conspiracy theories offer a further dimension interesting from a psychological standpoint. They offer the possibility to transfer one's value system into the social domain: According to Mason (1997), the moral self must learn to discern the values held by other persons and institutions; and should encourage others to act morally. Fivush and Buckner (1997) argue that language is not only a medium, but is both necessary to construct a self-concept and to engage in moral-based interaction with others. From this point of view, making stories that describe the ethics of institutions as well as one's own is not a possibility, but a necessity in moral development. Also, sharing these narratives is desirable.

A conspiracy theory, thus, could be seen as a differentiated story of our beliefs and values helping us to understand and express our non-conscious moral feelings. Historic or contemporary events and developments which threaten these values may become the initial nucleus for such a story. The need to construct such a story arises from living in a society where the generally acknowledged goal of individuation is no longer a mere adoption of common beliefs, but where becoming individual is the preferred goal.

Furthermore, a conspiracy theory would allow us to share our beliefs with others and to make us (and others) cautious about the violation of ethical standards. Horstmann (2012) even hypothesizes that apocalyptic scenarios, for example about World War III, are the main reason such scenarios have not become reality so far. Narratives about dystopian developments make us aware of such developments in the first place.

Likewise, in conspiracy theories such scenarios are a common topic. This need not end in a feeling of helplessness, or, as Melley (2000) termed it, agency panic. A conspiracy theory might be considered as a remedy; yet, not only in the way described by Melley as a defense mechanism of individualism: social exchange about a supposed conspiracy is comforting and reassuring. Taking part in such language games requires a widely known story nucleus (e.g., the terrorist attacks of 9/11), so others recognize the importance of the story and can affiliate. We might consider a good conspiracy theory as a kind of interface to find like-minded people and to overcome alienation.

### The conspiracy code

So far, we have considered:

– The importance of stories, to be more precise, of mythical stories, in human history.
– The importance of stories that mirror a person's belief and value system as a means of individuation. They explicate what is good or bad and can be considered helpful to shape one's value system by organizing one's life experience in a meaningful way.
– The potential to find like-minded people by engaging in the active exchange of value-expressing stories.

However, the psychological importance of narratives does not explain: Why is a *conspiracy* theory a method of choice? A story about morale, i.e., what is right and wrong, will necessarily include both moral extremes. A rivalry between good and bad allows the storyteller to make clear which side he or she is on. However, it would be comforting if evil deeds are done by a manageable part of society, not by the majority; otherwise, one would cast himself an outsider. Additionally, these deeds must be concealed, too; in other respects, the majority of society would have noticed and would either approve of these deeds, or be indifferent. Both options would be discomforting.

Here we meet with existing approaches to the phenomenon of conspiracy theories. Many observed features suit perfectly with our assertions sketched here:

– An immunization against counter-evidence makes a narration invulnerable. If the story reflects a person's most important beliefs and values, it is quite understandable why immunization is desirable.
– Four people had selected the item alleging to a Jewish involvement. The result suggests that anti-Semitic beliefs were present in our sample; but four out of 30 people might indicate that xenophobia is not the heart of every conspiracy theory. Yet, a conspiracy is in need for conspirers. We acknowledge the danger that some people might rely upon existing stereotypes—e.g., prejudice about Jews or Muslims. Exploiting such biases would indeed be a result of, not a reason for, conspiracy theories. An exemption would be a person who holds racist beliefs as most important conviction. The whole theory would be built around these convictions then—and mirror the psychological motifs described by Moscovici (1987).
– A powerless and underprivileged person might be in need to understand why he or she has failed in life; that means him or her as a person, with beliefs about right and wrong. We indeed could expect him or her to construct a rather extreme narrative that mirrors the severity of his failure in life. Here, attributional mechanisms to uphold the belief in a just world (Lerner, 1980) would be relevant, as described by Farr (1987).

Of course, a highly immunized, racist, and extreme conspiracy theory stands out. It attracts the attention of society and, consequently, of psychologists—and we indeed need to understand and explain the person behind such stories. In our sample, at least the one conspiracy theory we have stated in detail here would qualify for this extreme. But aside from this extreme shade of truth: there were 29 stories that demand deeper and more differentiated psychological analysis. When we regard conspiracy theories as a continuum of identity-shaping potential, the phenomenon is demystified. This will be an important step toward the appreciation “to what extent conspiracy theories reflect everyday cognitions” (Swami and Coles, 2010, p. 563).

This, of course, does not render research on individual differences useless. Actually, this specific research is highly relevant for our approach. Stories in general—and life stories in particular—are highly intertwined with a storyteller's personality. We assume that individual values determine the content of a conspiracy theory. We further assume that personality moderates if this theory is, for example, open for new evidence or highly immunized. Consequently, we suggest to further explore and analyze these interdependencies between *content* and *shape*. The mere *form*—a story about secret and potentially harmful deeds—would then be of lesser psychological relevance.

However, we must not neglect the fact of the harmful potential these theories bear. Considering them as an omnipresent and—in principle—benign psychological phenomenon helps us to explore why some people fall for extreme conspiratorial constructs of ideas which might lead to xenophobic or even racist arguments. It might also help us to understand how agitators deliberately use conspiracy theories to transport hateful ideology—wrapped up in a plausible plot that masks these foul intentions (Byford and Billig, 2001; Wood and Finlay, 2008). The question should not be: Why does one believe a racist *conspiracy theory*? Rather, we should ask: Why does one believe a *racist* conspiracy theory?

### Conflict of interest statement

The authors declare that the research was conducted in the absence of any commercial or financial relationships that could be construed as a potential conflict of interest.

## Footnotes

^1^Explained in more detail in a separate manuscript recently submitted to the same Frontiers Research Topic
